# Expression Profile of Urinary Exosomal miRNAs in Patients With Diabetic Kidney Disease and Their Association With Kidney Damage

**DOI:** 10.1155/ije/9927320

**Published:** 2025-09-23

**Authors:** Juan Li, Lulu Han, Ting Wang, Lin Yang, Hong Zhou

**Affiliations:** ^1^Department of Nephrology, The Second Hospital of Hebei Medical University, Shijiazhuang, Hebei, China; ^2^Department of Endocrinology, The First Central Hospital of Baoding, Baoding, Hebei, China; ^3^Department of Endocrinology, The Second Hospital of Hebei Medical University, Shijiazhuang, Hebei, China

**Keywords:** bioinformatic analysis, diabetic kidney disease, expression profile, microRNAs, urinary exosomes

## Abstract

**Purpose:** Diabetic kidney disease (DKD) is the primary cause of end-stage renal disease. The aim of this study is to identify noninvasive biomarkers for early-stage DKD or targets for DKD treatment through the analysis of urinary exosomal miRNA expression profiles in DKD patients.

**Methods:** The urinary exosomes were isolated from type 2 diabetes (T2DM) patients with DKD confirmed by renal biopsy (DKD-Exo). The urinary exosomal miRNAs expression profiles were detected using miRNA sequencing, and differentially expressed miRNAs were verified by real-time quantitative PCR. Target genes of these miRNAs and relevant pathways in DKD were analyzed by Gene Ontology (GO) and Kyoto Encyclopedia of Genes and Genomes (KEGG) enrichment. Human podocytes and renal tubular epithelial cells (TECs) were treated with DKD-Exo to investigate the effects of DKD-Exo on podocyte apoptosis and the epithelial–mesenchymal transition (EMT) of TECs.

**Results:** A total of 40 miRNAs were found to be differentially downregulated, 17 of which were named and 23 were untitled; miR-371a-3p, miR-371a-5p, miR-1260b, miR-222-3p, miR-1224-5p, and miR-1253 were reported in DKD for the first time. GO and KEGG pathway analyses suggest that these target genes are related to cellular apoptosis and renal fibrosis in DKD, and are involved in 135 pathways. *In vitro*, DKD-Exo induced the apoptosis of podocytes and collagen synthesis in TECs.

**Conclusion:** Our study implies that the urinary DKD-Exo could deliver biological information to podocytes or TECs, which play an important role in pathogenesis of DKD.

## 1. Introduction

Diabetic kidney disease (DKD) is a common microvascular complication in patients with diabetes and is the primary cause of end-stage renal disease (ESRD) around the world [1]. Despite glycemic control, it is difficult to prevent DKD progression. So, early diagnosis and timely treatment of DKD are very important. At present, the clinical diagnosis of DKD is mainly based on a progressive decline of estimated glomerular filtration rate (eGFR < 60 mL·min^−1^·1.73 m^−2^) or/and persistent increased urinary albumin creatinine ratio (UACR ≥ 30 mg/g) [2, 3], but these are nonspecificity, and it is required to take the diabetic course and fundus lesions into account. Also, kidney damage can occur even before urinary albumin and decline of renal function, such as glomerular hyperperfusion and hyperfiltration [4]. In necessity, renal biopsy is needed, and there is an urgent need to find the novel biomarkers and effective therapeutic targets for DKD at early stage. DKD is a complicated disease process; inflammation, oxidative stress, mitochondrial dysfunction, and impaired autophagy are involved in pathophysiologic mechanisms, eventually leading to kidney damage [2]. The pathological changes of DKD contain basement membrane thickening, mesangial cell hypertrophy, podocyte loss and apoptosis, and tubulointerstitial fibrosis [3]. Particularly, podocytes apoptosis and renal tubular epithelial cell (TEC) damage play an important role in the pathogenesis of DKD [4–6]. Due to requirement of high-energy and aerobic metabolism, renal tubules are more susceptible to the metabolic disturbances, leading to inflammation and fibrosis associated with DKD [7].

Exosomes are a kind of extracellular vesicles produced by various cells, which exist in all body fluids, including serum, urine, saliva, and so on. Exosomes can carry different molecular constituents of the donor cells, such as proteins, lipids, mRNAs, and microRNAs (miRNAs) into the recipient cells [8]. Because of double-layer membrane, exosomes are not easily degraded and can maintain signaling-molecule stability for long-distance transmission, and participate in cell–cell communication within the nephron [9–11]. Circulating exosomes are not filtered through the glomeruli, so urinary exosomes are thought to be secreted by natural cells of the urinary system, and can reflect pathophysiological changes in nephron under disease conditions. Therefore, urinary exosomes are likely derived from glomerular cells or renal TECs. MiRNAs are a type of noncoding RNAs, composed of roughly 22 nucleotide sequences, and are involved in the regulation of transcription or posttranscription of genes. Exosomes are abundant in miRNAs, and it is worth noting that exosomal miRNAs may have potential research value in the development of DKD. Emerging evidence suggests that exosomal miRNAs play vital roles in the occurrence of DKD by signal crosstalk among internal cells, mediating inflammation and autophagy in kidney [12–16]. Due to the superior stability and specificity of urinary exosomes, urinary exosomal miRNAs may become novel therapeutic targets for DKD.

Although some new biomarkers of DKD have been developed [17–19], the prognostic significance of these biomarkers is not specific to DKD. Recently, the application of high-throughput sequencing approaches to analyze biological samples has emerged as a strong tool in biomarker discovery. González-Palomo et al. observed the profile of urinary exosomal miRNAs in patients with T2DM and DKD, and found that the patients with DKD presented a significant increase in miR-126. Meanwhile, miR-146, miR-155, and miR-126, together with some clinical parameters, can predict the development of DKD [20]. Zang et al. affirmed differential expression of miR-21-5p and miR-30b-5p in individuals with DKD, and these miRNAs maybe become potential biomarkers associated with DKD [21].

In this study, we extracted urinary exosomes from T2DM patients with DKD and observed the expression features of urinary exosomal miRNAs, as well as analyzed relevant pathways by bioinformatics data. What counts is that these DKD patients all were confirmed by renal biopsy. Meanwhile, treatment of podocytes and renal TECs with these urinary exosomes confirmed the effects of urinary exosomes on podocyte apoptosis and epithelial–mesenchymal transition of TECs. We aimed to find a novel noninvasive biomarker or therapeutic target for DKD.

## 2. Materials and Methods

### 2.1. Data of Participants

Forty diabetic patients hospitalized in the Second Hospital of Hebei Medical University from February 2022 to October 2022 were selected, including 20 patients without DKD (DM group) and 20 patients with DKD (DKD group) confirmed by renal biopsy. Fifteen healthy volunteers were selected as the normal control (NC group), who came from the medical examination department of the same hospital. All specimens were obtained with the consent of the patients and the protocols were approved by the Clinical Research Ethics Committee of the Second Hospital of Hebei Medical University, the participants signed written informed consent forms, ethical review number: 2022-R059. The clinical data were collected, including gender, age, and body mass index (BMI). Fasting for 8 h, venous bloods of all participants were collected for biochemical indicators, including glycosylated hemoglobin (HbA1c), serum creatinine (SCr), triglyceride (TG), and total cholesterol (TC). First-morning urine (100 mL) was collected into sterile centrifuge tubes and then centrifuged at 3000 g for 30 min at 4°C to separate cells and debris. Subsequently, the supernatant was filtered through a 0.22-μm filter to remove bacteria and residual cells, and the resulting pellet was stored at −80°C for future use. In addition, the morning urine of each participant was collected for the measure of ACR. All samples were processed within 1 h of collection.

To generate an expression profile of urinary exosomal miRNAs, four individuals from the DM group and five from the DKD group were randomly selected for miRNA sequencing.

### 2.2. Extraction and Identification of Exosome

The separation of urinary exosomes is performed using ultracentrifugation. The urine samples (100 mL) were centrifuged at 15,000 g for 20 min at 4°C. The supernatant was aliquoted into 10 mL ultracentrifuge tubes and centrifuged at 200, 000g for 1 h at 25°C. Then, the supernatant was discarded, and 8 mL of PBS was added into the tubes to resuspend and centrifuged at 200, 000g for 1 h at 25°C. The above steps were repeated until all specimens were centrifuged. The pellet was suspended in 200 μl of isolation solution and frozen at −80°C. The exosome samples were diluted 5 times with PBS and then applied to 200-mesh nickel grids. Samples were stained with 2% phosphotungstic acid for 5 min at room temperature and air-dried. The exosomes were identified by transmission electron microscope analysis at Shanghai Umibio Biotechnology Co Ltd. The sizes and concentration distributions of these particles were measured by nanoparticle tracking analysis (NTA) (Particle Metrix, Germany). The exosomal surface markers CD63 and CD9 were detected via Western blot. The exosomes from the NC, DM, and DKD groups were, respectively, defined as the NC-Exo, DM-Exo, and DKD-Exo.

### 2.3. miRNA Sequencing

**Figure 1:**
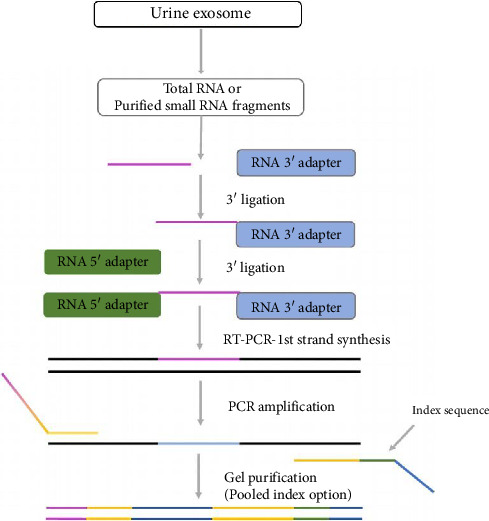
The miRNA sequencing experiment procedure.

The miRNA sequencing experiment procedure is shown in Figure 1. miRNA sequencing was completed using the Illumina sequencing platform. Sequencing data quality control primarily includes raw data statistics and quality control data statistics. The experimental procedures were conducted by Kangchen Biotech Co., Ltd. (Shanghai, China) as follows: Total RNA was extracted from tissues using TRIzol Reagent, according to Invitrogen's instructions. Degradation and contamination were assessed on 1% agarose gels, and RNA concentration was measured using the ND-2000 spectrophotometer (NanoDrop Technologies). RNA integrity was subsequently assessed using a 2100 Bioanalyzer (Agilent Technologies, Santa Clara, CA, USA). A total of 3 μg of RNA was used for the preparation of small RNA library for each sample. Sequencing libraries were generated using the NEBNext Multiplex Small RNA Library Prep Set for Illumina (NEB, USA), following the manufacturer's recommendations and index codes were added to each sample. LongAmp Taq 2X Master Mix, SR Primer for Illumina, and index (X) primer were used for PCR amplification. The quality of library was evaluated using the Agilent Bioanalyzer 2100 system with DNA High Sensitivity Chips. Following cluster generation, the libraries were sequenced on an Illumina platform, generating 50-bp single-end reads. miRNAs originate from precursors, which have a characteristic hairpin structure that can be used to predict novel miRNAs. miRNAs that cannot be aligned with Rfam and miRBase are aligned to the reference genome, and their surrounding sequences are extracted for secondary structure prediction using software miRDeep2. Based on the prediction results, miRNAs are identified by filtering with features such as Dicer cleavage site information and energy values. The expression levels of miRNAs were quantified using the transcripts per million reads (TPM) method. Significantly differentially expressed (DE) miRNAs were identified using DEseq2, with criteria of |log2FC| > 1 and FDR < 0.05. Sequencing of 9 samples was completed, yielding a total of 103.31 million raw reads, with each sample having over 10.33 million raw reads, and the percentage of Q30 bases exceeding 73.19%. Differential analysis of miRNAs was performed using DESeq2, DEGseq, and edgeR.

### 2.4. Real-Time Quantitative Polymerase Chain Reaction (RT-qPCR)

In this study, a total of 17 known differential miRNAs were screened, and 4 miRNAs were selected for validation. Total RNA was extracted with RNA-easyTM Isolation Reagent (Vazyme, Nanjing, China). cDNA was synthesized using the Bulge-LoopTM miRNA RT-qPCR Starter Kit (RiboBio, Guangzhou, China). RT-qPCR was conducted on a CFX96 PCR System (Bio-Rad, USA) using GoTaq qPCR Master Mix (Promega, USA). The relative expression of miRNAs was normalized using the endogenous reference miR-16-5P, and quantified by the 2^−ΔΔCt^ method. Primers were as follows:

• miR-371a-3p: Forward: 5′-ACUCAAACUGUGGGGGCACU-3, Reverse: 5′-AAGUGCCGCCAUCUUUUGAGUGU-3′;
• miR-483-5p: Forward: 5′-GCGAAGACGGGAGGAAAGA-3′, Reverse: 5′-AGTGCAGGGTCCGAGGTATT-3′;
• miR-124-3p: Forward: 5′-CGUGUUCACAGCGGACCUUGAU-3′, Reverse: 5′-UAAGGCACGCGGUGAAUGCCAA-3′;
• miR-371a-5p: Forward: 5′-ACUCAAACUGUGGGGGCACU-3′, Reverse: 5′-AAGUGCCGCCAUCUUUUGAGUGU-3′;
• miR-16-5p: Forward: 5′‐ ATAGAATCCTTGTATTATTATGTTTGGAC‐3′
• Reverse: 5′‐ ATAGGATCCAAATTATACTAGCAGGA‐3′

### 2.5. Target Gene Analyses of Differential miRNAs

Target gene predictions for miRNAs were conducted using the miRanda algorithm (https://www.miranda.org/). Predicted target genes were aligned using BLAST (https://blast.ncbi.nlm.nih.gov/) and annotated using the GO (https://www.geneontology.org/) and KEGG (https://www.genome.jp/kegg/) databases. Functional-enrichment analyses were conducted to identify targets significantly enriched in GO terms and metabolic pathways, with a Bonferroni-corrected *p*-value ≤ 0.05, compared to the whole-reference gene background. GO functional enrichment and KEGG pathway analyses were conducted using Goatools (https://github.com/tanghaibao/Goatools) and KOBAS (https://kobas.cbi.pku.edu.cn/home.do).

Through enrichment and pathway analysis, apoptosis and fibrosis pathways were found to be particularly prominent. Further literature analysis was conducted to assess the effects of urinary exosome intervention on podocyte apoptosis and renal tubular interstitial fibrosis.

### 2.6. Cell Culture

Both human tubular epithelial cells (HK-2) and human podocytes (HPCs) were purchased from ATCC (American Type Culture Collection). HPCs were cultured in 5.5 mM D-glucose RPMI-1640 (Gibco, USA) medium containing 10% fetal bovine serum (Gibco, USA) and 0.5% penicillin/streptomycin 100X (Solarbio, China), and then incubated in a humidified incubator at 37°C with 5% CO2. HK-2 cells were cultured in average DMEM and DMEM-F12 medium (DMEM; DMEM-F12, Gibco, USA), containing 10% fetal calf serum (Gibco; Thermo Fisher Scientific, Inc.), 10% L-glutamine, 0.5% penicillin/streptomycin, with 5% CO_2_ at 37°C. When cell fusion reached about 60%, the cells were respectively treated with NC-Exo, DM-Exo, and DKD-Exo, namely, the NC-Exo, DM-Exo, and DKD-Exo groups.

### 2.7. Exosome Uptake Assay

To observe whether HPCs and HK-2 cells can uptake urinary exosomes from the three groups, exosome suspension was labeled with diluent C solution (50 μL, Life Technologies) and PKH67 (4 μL, Sigma-Aldrich Corp., USA), incubated for 10 min at room temperature, and resuspended in PBS. HK-2 cells and podocytes were respectively treated with PHK67-labeled exosomes for 3 h, 7 h, and 24 h, the uptake of exosomes was measured by using an immunofluorescence microscope (Nikon Instruments, Tokyo, Japan), and the pictures were taken using a Nikon inverted fluorescence microscope (Eclipse TE200 microscope).

### 2.8. Cell Viability and Apoptosis of Podocytes

Cell viability was analyzed by cell counting kit (CCK-8, Dojindo, Japan) according to the manufacturer's instructions. The HPCs in logarithmic growth phase were collected, inoculated into a 96-well plate and incubated for 24 h, and then were treated respectively with NC-Exo, DM-Exo, and DKD-Exo in three groups, each consisting of six replicate wells. Urinary exosome concentrations were at 20 μg/μL, 40 μg/μL, 60 μg/μL, and 80 μg/μL. Following a 48-h incubation period, 10 μL of CCK-8 reagent was added to each well of the 96-well plate under sterile conditions; this was followed by an additional 4-h cell incubation. Optical density (OD) values for each group were measured at a wavelength of 450 nm using an automated microplate reader (Molecular Devices Corporation). Measurements were performed at least three times to obtain an average OD value for analytical calculations.

Apoptosis was analyzed using PE Annexin V/7-ADD detection kits. HPCs were treated respectively with NC-Exo, DM-Exo, and DKD-Exo in three groups for 48 h, and were thrice washed with PBS, incubated with trypsin devoid of EDTA, then examined microscopically for round cell morphology prior to terminating digestion with fetal bovine serum-containing cell culture medium. HPCs were harvested into EP tubes at a density of 1 × 10^5^ cells/tube and subjected to two rounds of centrifugation with precooled PBS. After the addition of 1 × binding buffer to each tube, 5 μl of PE Annexin V and 7-ADD were gently pipetted into the tubes for mixing. The samples were incubated in the dark at room temperature for 15 min, followed by the addition of 400 μl 1 × binding buffer, thorough mixing, and transfer to flow cytometry tubes equipped with a filter. Apoptosis rates were determined within 1 h via flow cytometry (BD, Biosciences, USA), with subsequent data analysis conducted using FlowJo software.

### 2.9. Western Blotting Analysis

HPCs and HK-2 cells were separately treated with three groups of urinary exosomes at a concentration of 60 μg/mL, designated as the NC-Exo, DM-Exo, and DKD-Exo groups. Subsequently, the cells were cultured in a normal medium for 48 h. Total proteins were isolated from HPCs, HK-2 cells, and exosomes by radio immunoprecipitation assay (RIPA) lysis buffer (Solarbio, China); concentrations were determined with a BCA protein analysis kit. 30 μg of denatured proteins were separated by 10% SDS-polyacrylamide gel electrophoresis (PAGE) (Epizyme, China) in each sample and then were transferred onto polyvinylidene fluoride (PVDF) membranes (Millipore, USA). The membranes were blocked with 5% skim milk (BioFroxx, Germany) for 2 h at 24°C before being incubated with primary antibodies: CD63 (1:1000, Abcam), CD9 (1:1000, Abcam), Bax (1:1000, Protein Tech), Bcl-2 (1:1000, Protein Tech), cleaved caspase-3 (1:500, Cell Signaling), *α*-SMA(1:1000, Protein Tech); Collagen I (1:500, Immunoway), Fibronectin (1:2000, Immunoway), *β*-actin (1:1000, Protein Tech), GAPDH (1:5000, Abcam) at 4°C overnight. The following day, membranes were incubated with horseradish peroxidase (HRP)-labeled goat anti-rabbit or anti-mouse secondary antibodies (1:5000, Affinity) for 1 h at 24°C. Protein bands were detected using chemiluminescence (ECL) reagents (General Electric, USA) and quantified with ImageJ software (Bio-Rad, USA).

### 2.10. Statistical Analysis

Statistical analysis was performed using Prism (GraphPad 8.0 Software). Normally distributed data are expressed as mean ± standard deviation (SD) and compared using an independent sample *t*-test, whereas non-normally distributed data are presented as the median with the 25th and 75th percentiles (interquartile range) and compared using the Mann–Whitney *U* test. The SNK method was used to analyze the data between the two groups. The data in multiple groups were analyzed using one-way analysis of variance (ANOVA), followed by Tukey's test. A level of *p* < 0.05 was identified statistically significant.

## 3. Results

### 3.1. Clinical Data of Participants

As shown in Table 1, HbA1c levels of the DM and DKD groups were observably higher than that of the NC group, and HbA1c levels of the DKD group were significantly higher than that of the DM group. Compared with the NC and DM groups, eGFR, SCr, TC, TG, and urine ACR were significantly increased in the DKD group. There were no differences in age, gender, BMI, and UA among the three groups.

**Figure 2:**
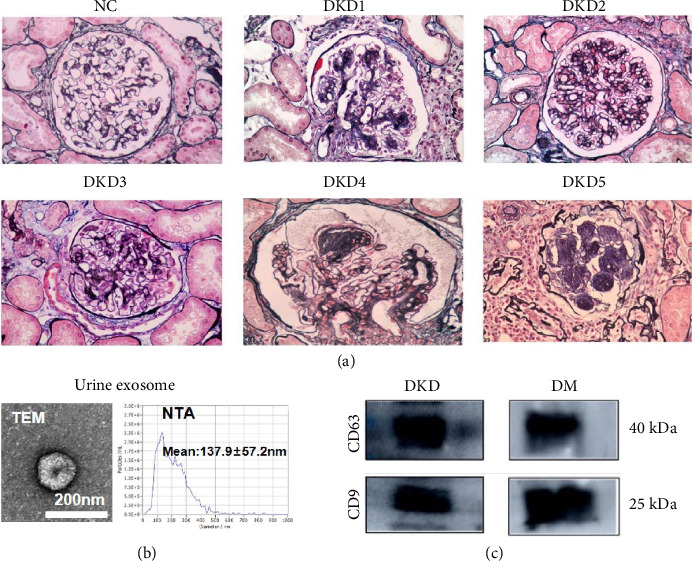
Renal pathology and characterization of urinary exosomes. (a) The structures of normal renal tissues from renal tumor patients without DKD and the renal tissues of DKD patients (PASM + MOSSON × 400). (b) Exosomes were visualized by transmission electron microscopy (TEM) (magnification: ×30, 000; scale bar: 200 nm). Particle size and concentration were measured using nanoparticle tracking analysis (NTA). And diameter sizes and their concentration distributions of exosomes were measured by nanoparticle tracking analysis (NTA). (c) The exosomal surface markers CD63 and CD9 were detected by Western blotting. Note: NC: normal control; DKD: diabetic kidney disease group; DM: diabetes mellitus group.

The structures of normal renal tissues from renal tumor patients without DKD and the renal tissues of DKD patients are shown in Figure 2(a). Compared with normal glomeruli, there were many changes of renal pathology in DKD patients, such as glomerular basement membrane thickening, mesangial cell and stromal hyperplasia, and inflammatory cell infiltration. The renal pathology of 5 DKD patients was manifested from proliferation of the mesangium and stroma to the formation of K-W nodules with tubular atrophy and interstitial cell infiltration.

### 3.2. Urinary Exosome Features and Identification

TEM presented that these vesicles appeared cup-shaped hemispherical structure with a double-layer membrane, their diameters ranged from 40 to 100 nm, the most were approximately 80 nm. NTA was used to identify the size of urinary exosomes. The mean diameter was 137.9 ± 57.2 nm. Western blotting showed that these vesicles expressed CD63 and CD9 proteins, suggesting that exosomes were successfully extracted from participants' urinary samples (Figures 2(b) and 2(c)).

### 3.3. Expression Characteristics of Exosomal miRNAs

**Figure 3:**
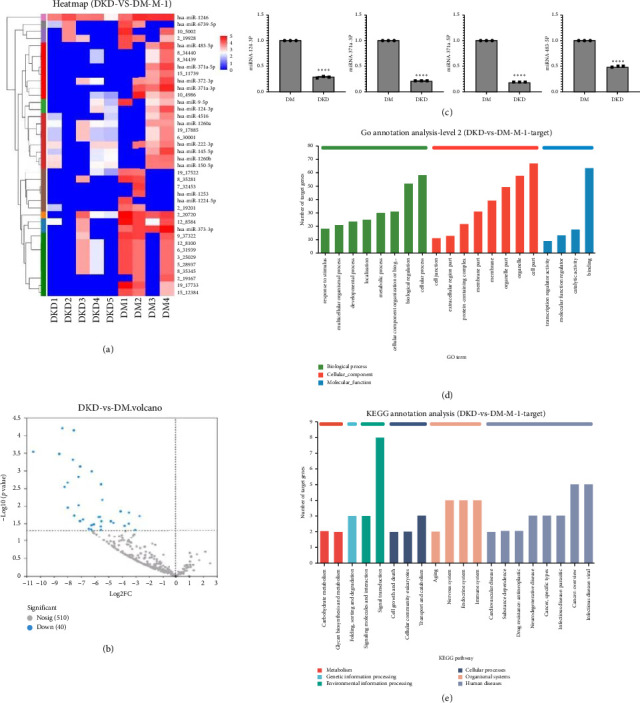
The miRNA expression profiles in urine exosome. (a) Heat map of differentially expressed miRNAs. Red color represents high relative expression level, and blue color represents low relative expression level. (b) Volcano plot of differentially expressed miRNAs. The blue dots represent the downregulated differential miRNAs. (c) Validation of four differentially expressed miRNAs by real-time PCR (*N* = 3). ^∗∗∗∗^*p* < 0.001 vs. DM group. (d) GO analysis revealed enrichment in biological processes, molecular function, and cellular component. (e) KEGG pathway analysis indicated associations with PI3K/Akt, MAPK, and p53 signaling pathways. Note: DM: diabetes mellitus exosomes; DKD: diabetic kidney disease exosomes.

The hierarchical clustering of DE miRNAs was shown by heat map and volcano plot (Figures 3(a) and 3(b)), and a total of 40 differential miRNAs were recorded, of which 17 were known and 23 were newly discovered in this study. These known miRNAs were downregulated in the DKD group, including miR-371a-3p, miR-483-5p, miR-373-3p, miR-9-5p, miR-145-5p, miR-372-3p, miR-371a-5p, miR-1260b, miR-222-3p, miR-1224-5p, miR-1246, miR-124-3p, miR-4516, miR-150-5p, miR-6739-5p, miR-1253, and miR-1260a. The miRNAs were also shown in descending order according to fold changes (Table 2). To validate the significant changes of miRNA expression, 4 of the most DE miRNAs were assessed by real-time qPCR (Figure 3(c)). The results showed that miR-371a-3p, miR-371a-5p, miR-483-5p, and miR-124-3p were downregulated in DKD group, consistent with the results of heat map.

### 3.4. GO Enrichment and KEGG Pathway Analyses

GO analysis showed that the target genes of these downregulated miRNAs were related to biological processes, molecular function, and cellular component. KEGG pathway analysis showed that these target genes were involved in 135 pathways, including Toll-like receptor, MAPK, PIK3/Akt, p53, and calcium signaling pathway as well as FOXO and apoptosis signaling pathways, etc. (Figures 3(d) and 3(e)). The pathways associated with cell apoptosis include Bcl-2 binding component 3 and PI3K/Akt signaling pathways. The pathways related to fibrosis include NF-κB and MAPK signaling pathways.

### 3.5. Exosome Uptake

**Figure 4:**
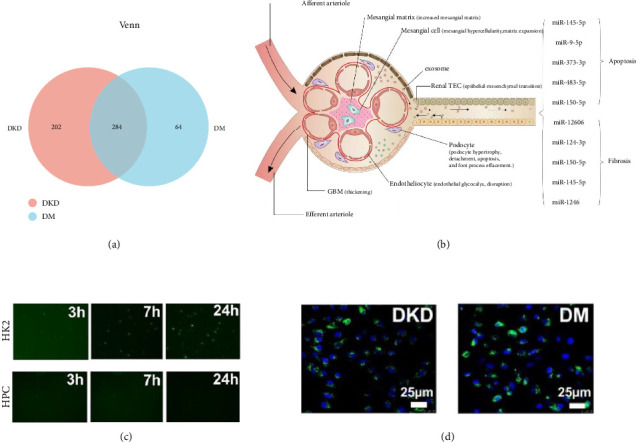
(a) Venn analysis showed that there were 284 miRNAs common to both the DM and DKD groups, with 202 miRNAs specific to the DKD group and 64 miRNAs specific to the DM group. (b) Schematic illustration of DKD-related glomerular changes, including GBM thickening, mesangial expansion, and podocyte foot process effacement. Exosome-mediated communication is proposed to contribute to fibrosis and apoptosis. (c and d) Immunofluorescence microscope observed the uptake of exosome in HPC and HK-2.

The Venn analysis between the DM and DKD sample groups shows the number of shared and unique miRNAs, as illustrated in Figure 4(a). A schematic model illustrating the role of DE miRNAs in DKD pathogenesis, with a particular emphasis on urinary exosomal miRNAs, is shown in Figure 4(b).

To observe whether HPCs and HK-2 cells could take up exosomes, exosomes were labeled with PKH67. From the treatment of podocytes and HK-2 cells with exosomes for 3 h, 7 h, and 24 h, it was observed that these cells began to uptake exosomes at 3-h point, and an absorption peak at 24-h point. It was shown that exosomes were located in the cytoplasm by laser confocal microscopy (Figures 4(c) and 4(d)).

### 3.6. Effects of DKD-Exo on the Viability and Apoptosis of Podocytes

**Figure 5:**
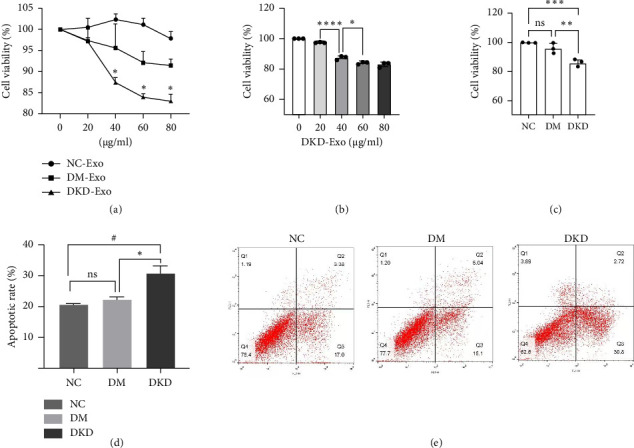
Effects of DKD-Exo on the viability and apoptosis of HPCs. (a–c) HPCs were treated with urinary exosomes (0–80 μg/mL) for 48 h. Cell viability was measured using CCK-8 assay. *N* = 3, ^∗^*p* < 0.05 vs. DM, ^#^*p* < 0.05 vs. NC. (d and e) Apoptosis was evaluated using annexin V/7-AAD staining and flow cytometry. Note: NC: normal control exosomes; DM: diabetes mellitus exosomes; DKD: diabetic kidney disease exosomes.

Compared with that of the NC-Exo and DM-Exo groups, the cell viability of HPCs treated with DKD-Exo significantly decreased, but there were no differences in the cell viability of podocytes between the NC-Exo and DM-Exo groups (Figure 5(a)). Moreover, the cell viability in the DKD-Exo group was significantly reduced at concentrations of 40 μg/mL, 60 μg/mL, and 80 μg/mL compared with the NC-Exo and DM-Exo groups; however, no significant differences in cell viability were found between the concentrations of 60 μg/mL and 80 μg/mL in DKD-Exo group. Therefore, a concentration of 60 μg/mL of urinary exosomes is considered feasible for this study (Figures 5(b) and 5(c)).

**Figure 6:**
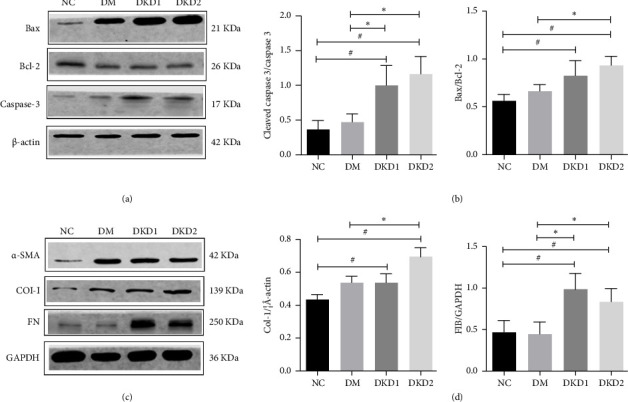
Effects of DKD-Exo on the apoptosis of HPCs and fibrosis of HK-2 cells. (a and b) Western blot analysis of bax, Bcl-2, and cleaved caspase-3 in podocytes. (c and d) Fibrosis markers (α-SMA, collagen I, FN) were analyzed in HK-2 cells. *N* = 3, ^∗^*p* < 0.05 vs. DM, ^#^*p* < 0.05 vs. NC. Note: NC: normal control exosomes; DM: diabetes mellitus exosomes; DKD: diabetic kidney disease exosomes. Abbreviations: Bax, Bcl-2-associated X protein; Bcl-2, B-cell lymphoma 2; *α*-SMA, alpha smooth muscle actin; FN, fibronectin; COI-I, collagen I; GAPDH, glyceraldehyde 3-phosphate dehydrogenase.

Flow cytometry results showed that a significant increase in apoptosis rate of HPCs in the DKD-Exo group when compared with the DM-Exo and NC-Exo groups (Figures 5(d) and 5(e)). Compared with the NC-Exo and DM-Exo groups, the ratio of Bax to bcl-2 and the expression of cleaved caspase-3 were observably increased in the DKD-Exo group (Figures 6(a) and 6(b)).

### 3.7. Effects of DKD-Exo on Collagen Synthesis and Trans-Differentiation of HK-2 Cells

Compared with the NC-Exo and DM-Exo groups, the levels of Collagen I and FN proteins were significantly increased in the DKD-Exo group, but there were no differences in the levels of Collagen I and FN proteins between the NC-Exo and DM-Exo groups. Compared with the NC-Exo group, the levels of *α*-SMA protein were significantly increased in the DM-Exo and DKD-Exo groups, but there were no differences in the levels of *α*-SMA protein between the DM-Exo and DKD-Exo groups (Figures 6(c) and 6(d)).

## 4. Discussion

DKD is one of the most common chronic complications of DM, which increases the disability and mortality in patients with DM. Up to now, no specific method has yet been established to restrain the progression of DKD.

Exosomes are a kind of nanovesicles derived from various mammalian cells, and can be present in almost all biological fluids, such as blood, urine, and interstitial fluids. Exosomes are abundant in miRNAs; these miRNAs hoarded within exosomes are more stable and more resistant to degradation than mRNAs [22, 23]. Exosomal miRNAs stem from different donor cells and possess specific expression profiles of miRNAs, also exosomal miRNAs can be transferred to target cells and mediated cell-to-cell communication through the delivery of biological information, thereby modulating the pathophysiological processes of the human body, and reflecting pathological changes in cells or tissues under disease conditions [24–26]. It is reported in vitro that exosomes could shuttle among kidney cells and the exosomal miRNAs from impaired renal cells could cause further aggravation of kidney damage from glomeruli to tubules [27]. Because the circulating exosomes secreted by the different cells in the body cannot cross the glomerular filtration barrier, urinary exosomes are typically secreted by renal epithelial cells downstream of the glomerular barrier, such as podocytes, and epithelial cells from the proximal and distal tubules and collecting ducts [28, 29]. Due to the superior stability and specificity of urinary exosomal miRNAs, the changes in miRNAs of urinary exosomes could indicate the progression of DKD [5, 30–33]. In the study, we observed the expression profiles of urinary exosomal miRNAs in DKD patients diagnosed by renal biopsy, and a total of 40 miRNAs were found to be differentially downregulated, 17 named miRNAs included miR-371a-3p, miR-483-5p, miR-373-3p, miR-9-5p, miR-145-5p, miR-372-3p, miR-371a-5p, miR-1260b, miR-222-3p, miR-1224-5p, miR-1246, miR-124-3p miR-4516, miR-150-5p, miR-6739-5p, miR-1253, and miR-1260a; 23 miRNAs were untitled. The study indicates that miRNAs implicated in apoptosis include miR-483-5p, miR-9-5p, miR-145-5p, miR-150-5p, and miR-373-3p, whereas those associated with fibrosis include miR-150-5p, miR-124-3p, miR-1260b, miR-145-5p, and miR-1246. Some DE miRNAs in our study were not reported in DKD patients, such as miR-371a-3p, miR-371a-5p, miR-1260b, miR-222-3p, miR-1224-5p, and miR-1253.

Recent studies have shown that miR-483-5p is involved in renal TEC injury and interstitial fibrosis. Liu et al. confirmed that circulating miR-483-5p was significantly downregulated in DKD patients, which could distinguish DKD from T2DM patients [34, 35]. Su et al. analyzed miRNA expression profiles in kidney from DKD patients through miRNA microarray and found that expression of miR-483-5p in renal tissues was observably downregulated [36]. This study demonstrates that miR-483-5p is downregulated in urinary exosomes, as evidenced by validation and *in vitro* cell experiments, which may contribute to podocyte apoptosis and tubular fibrosis. Consistent with previous findings, miR-483-5p may serve as a noninvasive biomarker for the diagnosis of DKD. It is reported that downregulation of miR-150-5p in HG-treated HK-2 cells was involved in tubular damage in DKD by suppressing mitophagy, and DRP1 was a target gene of miR-150-5p [37]. These results were consistent with our findings in urinary exosomes. Numerous studies are in favor of serum miR-130b and miR-21 and urinary miR-192, let-7c-5p, miR-29c-5p, and miR-15b-5p as potential diagnostic biomarkers for DKD [38–41]. It is reported that 4 DE serum miRNAs, including miR-21-3p, miR-378-3p, miR-16-5p, and miR-29a-3p, are closely correlated with the pathological grade of patients with DKD [42]. Meng et al. reported that miR-372-3p serves as a potential diagnostic marker for DKD and influences high glucose–induced dysfunction in glomerular endothelial cells through its interaction with fibroblast growth factor-16 [43].

Hyperglycemia induced nephritic inflammation, oxidative stress, apoptosis, autophagy, epithelial–mesenchymal transition, and fibrosis, which contribute to the occurrence and development of DKD. Through GO and KEGG analysis, we found that the target genes of these urinary exosomal miRNAs were related to biological processes, molecular function, and cellular component, and were involved in apoptosis and fibrosis signaling pathways. Podocyte loss and apoptosis as well as renal TEC damage are thought to play a critical role in the pathogenesis of DKD, especially the renal interstitial fibrosis contributes to the deterioration of DKD [44, 45]. Our *in vitro* experiments showed that DKD-Exo mediated apoptosis of podocytes and increased collagen synthesis in TECs, which suggests that there is a cell interaction between podocytes and TECs through exosomes. These are involved in the pathological process of DKD. Zhang et al. found that miR-124 expression was dramatically downregulated in renal tissues from STZ-induced diabetic mice and in HK-2 cells treated with HG and miR-124 might lead to renal fibrosis through TLR4/NF-κB pathway [46]. Different from our results, Wang et al. showed that miR-145-5p was upregulated in HG-induced HK-2 cells, and dual-specificity phosphatase 6 (DUSP6) was identified as a target gene of miR-145-5p. MiR-145-5p/DUSP6 axis plays an important role in the inflammatory response and fibrosis of HG-stimulated HK-2 cells, knockdown of miR-145-5p alleviated renal injury in DKD mice [47]. But Wei et al. indicated that miR-145-5p was downregulated in podocytes in HG conditions. miR-145-5p overexpression attenuated HG-induced podocytes apoptosis by targeting the Notch signaling pathway [48]. Zhou et al. showed that miR-145-5p expression was remarkably downregulated in HG-induced HK-2 cells, which mediated HG-induced oxidative stress, inflammation, and fibrosis in HK-2 cells by targeting VASN [49]. This study systematically identified DE miRNAs in urinary exosomes derived from DM and DKD. Previous *in vitro* and *in vivo* studies have demonstrated that these miRNAs play a crucial role in cellular proliferation, podocyte apoptosis, and renal fibrosis in DKD. Through the analysis of readily available clinical urine specimens, this investigation offers novel perspectives for the development of noninvasive clinical biomarkers.

The limitations of this study are lack of some validation *in vivo* and *in vitro* under HG conditions. The numbers of whose gene sequencers are performed are small, and further research is needed to confirm this hypothesis. In addition, the study employs a cross-sectional design and lacks longitudinal clinical outcomes necessary to establish prognostic value.

To sum up, our study indicates that there are DE miRNAs in urinary exosomes from DKD patients, bioinformatics analysis shows that these downregulated miRNAs are involved in apoptosis and fibrosis signaling pathways. Treatment of podocytes and TECs with DKD-Exo showed increased cell apoptosis and collagen synthesis *in vitro*, which suggests that changes of urinary exosomes from DKD patients may provide noninvasive biomarkers or therapeutic targets for DKD.

## Figures and Tables

**Table 1:** Characteristics of participants.

*N*	NC 15	DM 20	DKD 20
Age (years)	55.33 ± 5.26	54.85 ± 0.91	57.05 ± 5.19
Sex (female/male)	7/8	10/10	10/10
BMI (kg/m^2^)	25.382 ± 17	24.682 ± 0.17	24.86 ± 1.97
ACR (mg/g)	7.45 ± 4.21	10.15 ± 6.13	5443.8 ± 1940.28^∗∗^
SCr (μmol/L)	66.726 ± 91	61.75 ± 12.46	120.1 ± 43.42^∗∗^
eGFR (mL/min)	100.78 ± 7.40	103.85 ± 13.65	59.15 ± 19.69^∗∗^
UA (μmol/L)	335.06 ± 0.22	345 ± 54.28	376.75 ± 73.09
TC (mmol/L)	4.53 ± 0.43	4.54 ± 0.56	5.15 ± 0.59^∗∗^
TG (mmol/L)	1.155 ± 0.30	1.12 ± 0.42	1.36 ± 0.28^∗∗^
HbA1c (%)	5.5 ± 0.15	8.4 ± 2.35^#^	9.53 ± 1.49^∗∗^

*Note:* The data are expressed as the mean ± SEM, ^*#*^*p* < 0.05 vs. NC group; ^∗∗^*p* < 0.01 vs. DM and NC groups. ACR, Urinary albuminuric creatinine ratio; eGFR: glomerular filtration rate; UA, serum uric acid; TG, triglycerides; HbA1c, glycosylated hemoglobin.

Abbreviations: BMI = body mass index, Scr = serum creatinine, TC = total cholesterol.

**Table 2:** Known miRNAs with notable differential expression.

Mature-id	DKD/DM fold change	DKD/DM *p* value	Regulation
hsa-miR-371a-3p	0.003	6.25E − 05	Down
hsa-miR-483-5p	0.003	0.00033401	Down
hsa-miR-373-3p	0.007	0.000760455	Down
hsa-miR-9-5p	0.007	0.001490016	Down
hsa-miR-145-5p	0.022	0.002430467	Down
hsa-miR-372-3p	0.023	0.006679351	Down
hsa-miR-371a-5p	0.007	0.009622864	Down
hsa-miR-1260b	0.058	0.014072619	Down
hsa-miR-222-3p	0.089	0.015364562	Down
hsa-miR-1224-5p	0.006	0.018645958	Down
hsa-miR-1246	0.152	0.019286976	Down
hsa-miR-124-3p	0.035	0.027010338	Down
hsa-miR-4516	0.04	0.030078458	Down
hsa-miR-150-5p	0.088	0.031630516	Down
hsa-miR-6739-5p	0.013	0.035177044	Down
hsa-miR-1253	0.012	0.044285116	Down
hsa-miR-1260a	0.122	0.047376458	Down

## Data Availability

The datasets generated and analyzed during the current study are available in the Sequence Read Archive (SRA) database (PRJNA 1099669) (https://www.ncbi.nlm.nih.gov/).

## Nomenclature

DKD: Diabetic kidney disease
ESRD: End-stage renal disease
T2DM: Type 2 diabetes
TECs: Renal tubular epithelial cells
EMT: Epithelial–mesenchymal transition
GO: Gene Ontology
KEGG: Kyoto Encyclopedia of Genes and Genomes
eGFR: Estimated glomerular filtration rate
BMI: Body mass index
HK-2: Human tubular epithelial cells
HPCs: Human podocyte cells
